# Screening of metabolic-related biomarkers linking intervertebral disc degeneration and type 2 diabetes based on comprehensive bioinformatics analysis and machine learning

**DOI:** 10.1016/j.bbrep.2026.102593

**Published:** 2026-04-22

**Authors:** Ba Qiu, Tao Chen, Qi Hong, Xiaoxing Xie, Mintao Xue, Ning Xu, Changgui Shi, Guohua Xu, Weiheng Wang, Muhammad Sulaiman, Yanhai Xi

**Affiliations:** a Department of Orthopedics, Spine Surgery, Second Affiliated Hospital of Naval Medical University, Shanghai, 200003, China; b Department of Key Laboratory, The Affiliated Changshu Hospital of Xuzhou Medical School, The Second People's Hospital of Changshu, Changshu, Jiangsu, 215500, China; c National Key Laboratory of Natural Target Medicine, China Pharmaceutical University, Nanjing, 210009, China

**Keywords:** Intervertebral disc degeneration, Type 2 diabetes, BCAA, Machine learning, AMBP

## Abstract

**Background:**

Intervertebral disc degeneration (IVDD) is a prominent etiology of lower back pain. Type 2 diabetes (T2D), the most prevalent metabolic disorder, may expedite IVDD progression through mechanisms involving hyperglycemia, advanced glycation end products, and microvascular complications. We aim to identify the biomarkers for T2D and IVDD.

**Methods:**

We retrieved two datasets pertaining to IVDD and T2D respectively from the Gene Expression Omnibus (GEO) database. Initially, differential expression analysis was conducted to identify differentially expressed genes (DEGs) in each group. Subsequently, machine learning algorithms including Boruta algorithm, Random Forest (RF), Support Vector Machine (SVM), and Extreme Gradient Boosting (XGBoost) were utilized to explore co-biomarkers for IVDD and T2D by analyzing the intersection of DEGs sets between the two groups. The clinical diagnostic value of these biomarkers was evaluated using ROC curve. Additionally, we employed the CYBERSORT and Single-cell sequencing to investigate the infiltration of immune cells in two diseases. Finally, the expression of biomarkers and the correlation between BCAA and IVDD was experimentally validated.

**Results:**

AMBP, a shared biomarker of IVDD and T2D, were identified using differential analysis and machine learning algorithms. In addition, CYBERSORT analysis and single-cell sequencing results revealed significant correlations between AMBP and cellular immunity. Finally, experimental validation on clinical samples and HNPC cells confirmed upregulation of AMBP, and the correlation between BCAA metabolism and AMBP.

**Conclusion:**

AMBP, as the shared biomarker between IVDD and T2D, has great clinical diagnostic value, which therefore may be a potential regulatory factor for two diseases.

## Highlights

• AMBP identified as a key shared biomarker for both intervertebral disc degeneration and type 2 diabetes.
• AMBP shows strong diagnostic value and is closely linked to immune cell infiltration in both diseases.
• AMBP correlates with impaired BCAA metabolism**,** its reversal mitigates metabolic dysfunction in disc cells.
• Knockdown of AMBP reduces cellular senescence and inflammation, highlighting its therapeutic potential.

## 1 Introduction

Lower back pain is a prevalent musculoskeletal disorder which has emerged as the primary reason of disability worldwide [1]. It exerts a significant impact on individuals' occupational performance and daily functioning, leading to substantial socio-economic burdens. Intervertebral disc degeneration (IVDD) stands out as one of the principal etiologies for lower back pain, accounting for approximately 34-68% of cases [2].

The intervertebral disc is comprised of the nucleus pulposus (NP), annulus fibrosus (AF), and cartilaginous endplate (CEP), which serve to connect the spine, transmit and alleviate mechanical stress [3]. NP is comprised of NP cells, type II collagen and proteoglycans. AF provides support by wrapping around the nucleus pulposus between the vertebral bodies. AF consists of two layers: the inner layer primarily composed of chondrocytes and type II collagen fibers, while the outer layer mainly consisting of fibroblast-like cells and type I collagen fibers [4]. CEP, situated between the nucleus pulposus and vertebral body, is primarily consisting of type II collagen fibers and proteoglycans. NP exchange substances with vertebral bodies via capillary diffusion in the cartilaginous endplate [5]. The occurrence of IVDD is closely linked to genetic predisposition [6], mechanical stress [7], metabolic disorders [8] and senescence [9], etc. Considerable research has been conducted on factors such as inflammatory cytokines, mechanical stress, and senescence in relation to IVDD. However, limited attention has been given to the significance and mechanisms of metabolic disorders in IVDD.

Type 2 diabetes (T2D) is a worldwide metabolic disorder, representing 90% in all diabetes [10]. T2D is characterized by pre-existing hyperinsulinemia, insulin resistance and subsequent β-cell dysfunction [11]. As widely recognized, microvascular disease stands out as the predominant complication of T2D. Notably, patients with T2D also exhibit an elevated incidence rate of orthopedic conditions including osteoporosis, pathological fractures [12], spinal stenosis [13]. Lots of researches have consistently revealed a significant link between T2D and IVDD [14]. A cross-sectional study, utilizing a comprehensive insurance database in the United States, further strengthens the evidence supporting the association between T2D and IVDD [15]. Similarly, a retrospective study conducted in India revealed that prolonged inadequate glycemic control among patients diagnosed with T2D constitutes a significant risk factor for IVDD [16]. Advanced glycation end products (AGEs) serve as crucial biochemical markers for T2D. Prolonged intake of AGEs leads to a decrease in intervertebral disc height and glycosaminoglycan content in the nucleus pulposus [17], alongside an upregulation of MMP6 production in NP tissue [18]. This subsequently triggers inflammasome-mediated inflammation [19], thereby exacerbating IVDD. Hyperglycemia levels can induce increased apoptosis of notochord cells within the rat nucleus pulposus [20]. The intervertebral disc is an avascular tissue, primarily obtaining nutrients through capillary permeation of the cartilaginous endplate [21]. The cartilage endplate of patients with T2D undergo narrowing and obstruction, resulting in ossification of the endplate. This further impedes nutrient supply to the intervertebral disc, leading to accumulation of toxic metabolites and increased apoptosis of nucleus pulposus cells within the disc [20].

Branched-chain amino acid (BCAA) takes up approximately 35% of the essential amino acids in mammals, comprising leucine, isoleucine, and valine. BCAA plays a pivotal role as a significant biomarker for various metabolic-related diseases [22]. Research findings indicate that there is an observed increase in circulating BCAA among patients with T2D and obesity, suggesting that the elevation of plasma BCAA not only contributes to insulin resistance but also serves as a robust predictor for the development of diabetes in the subsequent decade [23]. Although the precise role of BCAA in IVDD remains insufficiently investigated, the association between BCAA and senescence has emerged as a prominent area of scientific inquiry. Dietary restriction of BCAA has demonstrated an ability to prolong the lifespan of male mice with normal health, whereas prolonged supplementation with BCAA can induce obesity and reduce their lifespan by 10% [24]. Based on the association of IVDD with senescence and T2D, we speculate that excessive intake of BCAA may lead to the occurrence and progression of IVDD through some unknown mechanisms. Therefore, we conducted an assessment to determine the correlation between biomarkers and BCAA metabolism in IVDD.

In this study, we initially utilized differential genes expression analysis and four machine learning methods to identify shared biomarkers for IVDD and T2D, and assessing biomarkers’ clinical diagnostic value. Subsequently, we investigated the extent of immune cell infiltration in IVDD and T2D, as well as the correlation between biomarkers and immune cells. Finally, experimental validation was conducted to demonstrate the reliability of our research findings. Our aim is to provide an innovative perspective for treating IVDD and T2D through this study.

## 2 Methods

### 2.1 Collection and processing of genetic data

We retrieved two sets of IVDD (GSE186542, GSE167199) and T2D datasets (GSE20966, GSE25724) from the Gene Expression Omnibus (GEO) respectively. All datasets underwent batch correction using the 'SVA' package in R to generate two integrated datasets [25]. Ultimately, the IVDD integrated dataset comprised 6 control groups and 6 case groups, while the T2D integrated dataset included 17 control groups and 16 case groups.

### 2.2 Differentially expressed genes and gene set enrichment analysis

Differential expression analysis between case and control groups in the IVDD and T2D datasets was conducted using the LIMMA package in R, identifying differentially expressed genes (DEGs) with thresholds of |log_2_FC| > 1 and corrected P-value < 0.05^27^. Results were visualized through heatmaps and volcano plots, with downregulated genes in blue and upregulated genes in red. Gene set enrichment analysis (GSEA) was subsequently performed on DEGs from both datasets using R to characterize functional pathway alterations. Venn diagrams were utilized to illustrate the intersection of DEGs between these two diseases, while heatmaps displayed their expression levels in respective study cohorts.

### 2.3 Machine learning

We employed four machine learning algorithms, Boruta, SVM, RF and XGBoost, to conduct a comprehensive screening for common biomarkers associated with IVDD and T2D [26]. Boruta was utilized to assess the importance of features by employing randomization and repeated sampling techniques, thereby identifying the most influential variables that impact the target variable. RF was employed to generate new training samples through random selection with replacement from the original training set, demonstrating high efficiency, accuracy, and resistance against overfitting. SVM is suitable for our research because of its efficient processing ability of high-dimensional feature space. XGBoost has the characteristics of high efficiency, flexibility and effectiveness. The intersection genes of these four algorithms were considered as potential biomarkers for IVDD and T2D.

### 2.4 Evaluation of clinical diagnostic value

ROC curve was utilized to assess the clinical diagnostic efficacy of biomarkers in IVDD and T2D.

### 2.5 Analysis of immune cell infiltration

CIBERSORT is a tool that uses gene expression data and applies the deconvolution algorithm to evaluate the abundance of immune cell subsets in tissue samples [27]. Using the CIBERSORT algorithm with 1000 permutations, we employed a statistical significance level of p < 0.05 to predict the infiltration of immune cells in two sample groups. The analysis was performed using the LM22 signature matrix and quantile normalization disabled as recommended for RNA-seq data.

### 2.6 Single-gene GSEA

To enhance our comprehension of gene expression levels in highly enriched functional pathways, we conducted single-gene GSEA using the R software with gene set permutations set to 1000. [28]. The false discovery rate (FDR) was controlled using the Benjamini-Hochberg procedure, and an adjusted P value < 0.05 was considered statistically significant.

### 2.7 Single-cell analysis

We identified IVDD cells expressing more than 50,000 but less than 200,000 highly variable genes, and T2D cells expressing more than 25,000 but less than 100,000 highly variable genes. Subsequently, with mitochondrial genes less than 10% as the standard,we selected the top 2000 highly variable genes from each group for further analysis. Utilizing the UMAP algorithm [29], we successfully classified both samples into a comprehensive set of 21 distinct cell subtypes and analyzed the expression patterns of characteristic genes within each subtype. ALL cell types annotations were performed by single R package [30].

### 2.8 Clinical sample acquisition

The NP specimens in this study were obtained from 20 patients with IVDD (10 male, 10 female, mean age 52.3 ± 6.7 years). This investigation was approved by the Ethics Committee of the Second Affiliated Hospital of Naval Medical University (Approval No.2021SL044). Written informed consent was obtained from each participant. All experiments were conducted in accordance with the Declaration of Helsinki. Demographic characteristics of the patients are summarized in Table 1.

**Table 1:** Demographic and clinical characteristics of the study cohort.

Characteristic	Total Cohort (n = 20)	Grade I/II Group (n = 10)	Grade III/IV Group (n = 10)	p-value
Age (years), mean ± SD	53.2 ± 7.5	50.8 ± 6.9	55.6 ± 7.6	0.15
Sex, n (%)				1.00
Male	10 (50.0)	5 (50.0)	5 (50.0)	
Female	10 (50.0)	5 (50.0)	5 (50.0)	
Body Mass Index (kg/m^2^), mean ± SD	24.8 ± 3.2	23.9 ± 2.8	25.7 ± 3.4	0.22
Hypertension	6 (30.0)	2 (20.0)	4 (40.0)	0.63
Smoking History, n (%)	7 (35.0)	3 (30.0)	4 (40.0)	1.00
Fasting Plasma Glucose (mmol/L)	5.8 ± 1.2	5.5 ± 0.9	6.1 ± 1.4	0.28
HbA1c (%)	5.9 ± 0.8	5.7 ± 0.6	6.1 ± 0.9	0.31

### 2.9 Cell culture and transient transfection

Human nucleus pulposus cells (HNPC, 202511C) were purchased from Hefei Wanwu Biotechnology Co., LTD. (Hefei, China). The cell line was authenticated by short tandem repeat profiling and tested negative for mycoplasma contamination within three years prior to use. Cells were cultured in DEME F-12 medium with 10% FBS. The negative control (NC), AMBP siRNA was transfected into the cells with Lipofectamine 2000 (Invitrogen, USA). The target sequences for AMBP siRNA were TGCAGGAAAACTTCAATATCTCT (si-AMBP#1) and CAGGAAAACTTCAATATCTCTCG (si-AMBP#2).

### 2.10 Quantitative Real-Time Polymerase Chain Reaction (qRT-PCR)

cDNA was extracted from 500 ng RNA using HiScript II SuperMix (Vazyme, China).

qRT-PCR was performed using SYBR Green Master Mix in an ABI 7500 System (Thermo Fisher, USA). The PCR amplification conditions were 94 °C for 10 min, followed by 45 cycles of 94 °C for 10 s and 60 °C for 45 s. GAPDH was used as the internal reference. The primer pairs for the target genes are listed in Table 2.

**Table 2:** Sequences of primer pairs for the genes.

Gene	Forward primer sequence (5′-3′)	Reverse primer sequence (5′-3′)
SLC7A5	GCCACAGAAAGCCTGAGCTTGA	ATGGTGAAGCCGATGCCACACT
SLC25A44	GAGGCTATGTGGCTTCACTGCT	GACAATGTGAGGGCACTCCTTAG
BCAT1	GCTCTGGTACAGCCTGTGTTGT	TGCCAGCTTAGGACCATTCTCC
BCAT2	CGCTGAATGGTGTTATCCTGCC	CAGCAACTGCTTCATGGTGATCG
BCKDHA	CAGAGAACCAGCCCTTCCTCAT	AGCCTTGGCTCAGCAGATAGTG
BCKDHB	TGCACTGTTGGCTTGCGAGACA	TTCCGCAATGGCAGTAGCTCCA
P16	CTCGTGCTGATGCTACTGAGGA	GGTCGGCGCAGTTGGGCTCC
P21	AGGTGGACCTGGAGACTCTCAG	TCCTCTTGGAGAAGATCAGCCG
P53	CCTCAGCATCTTATCCGAGTGG	TGGATGGTGGTACAGTCAGAGC
IL6	AGACAGCCACTCACCTCTTCAG	TTCTGCCAGTGCCTCTTTGCTG
IL18	GATAGCCAGCCTAGAGGTATGG	CCTTGATGTTATCAGGAGGATTCA
IL4	CCGTAACAGACATCTTTGCTGCC	GAGTGTCCTTCTCATGGTGGCT
AMBP	CCGACAACATCCAAGTGCAGGA	GTGCTCACTGTCATCCTGTCCA
GAPDH	AATGGGCAGCCGTTAGGAAA	GCCCAATACGACCAAATCAGAG

### 2.11 Western blot

Total protein was extracted using RIPA buffer. Following quantification, equal protein amounts were electrophoresed on 10% SDS-PAGE gels and subsequently transferred to 0.45-μm PVDF membranes. Membranes were blocked for 2 h with 5% skimmed milk. Primary antibodies diluted in blocking buffer were then incubated overnight at 4 °C. The specific antibodies used were: anti-Phospho-P65 (82335-1-RR, Proteintech; 1:500), anti-P65 (80979-1-RR, Proteintech; 1:1000), anti-NLRP3 (68102-1-Ig, Proteintech; 1:1000), anti-P16 (10883-1-AP, Proteintech; 1:1000), anti-P21 (10355-1-AP, Proteintech; 1:1000), anti-P53 (60283-2-Ig, Proteintech; 1:1000), and anti-GAPDH (ab8245, Abcam; 1:5000). Following primary incubation, membranes were incubated with appropriate secondary antibodies for 1 h at room temperature. Protein bands were finally detected using an ECL chemiluminescence kit (Beyotime, Shanghai) and visualized on a Tanon 4600 imaging system (Tanon Science and Technology Co., Ltd.).

### 2.12 Enzyme-linked immunosorbent assay (ELISA)

Cytokine quantification was performed using a commercial double-antibody sandwich ELISA kit (Elabscience, E-EL-H0315, AMBP) according to the manufacturer's instructions. Briefly, a high-affinity microplate was pre-coated with specific capture antibody. Standards and test samples were added to the wells, followed by biotin-conjugated detection antibody. After incubation, captured cytokines formed complexes with both the solid-phase antibody and the detection antibody. Unbound components were removed by washing. Horseradish peroxidase (HRP)-labeled streptavidin (Streptavidin-HRP) was then added. Following another wash step, the chromogenic substrate 3,3′,5,5′-tetramethylbenzidine (TMB) was added and incubated in the dark to develop color. Color intensity, measured at 450 nm, was proportional to the cytokine concentration in the sample. The reaction was stopped using stop solution prior to absorbance measurement.

### 2.13 SA-β-gal assay

Cellular senescence was assessed using Senescence-Associated β-Galactosidase (SA-β-gal) staining (Senescence β-Galactosidase Staining Kit, #C0602, Beyotime, China), performed according to the manufacturer's protocol. SA-β-gal-positive cells were quantified via ImageJ software.

### 2.14 Statistical analysis

The R software (version 4.1.2) is utilized for conducting statistical analysis, with the results being presented as mean ± standard deviation. The comparison between the two groups is performed using an unpaired Student's t-test, and a P-value below 0.05 indicates statistical significance.

## 3 Results

### 3.1 Data processing and differential gene expression analysis

**Fig. 1:**
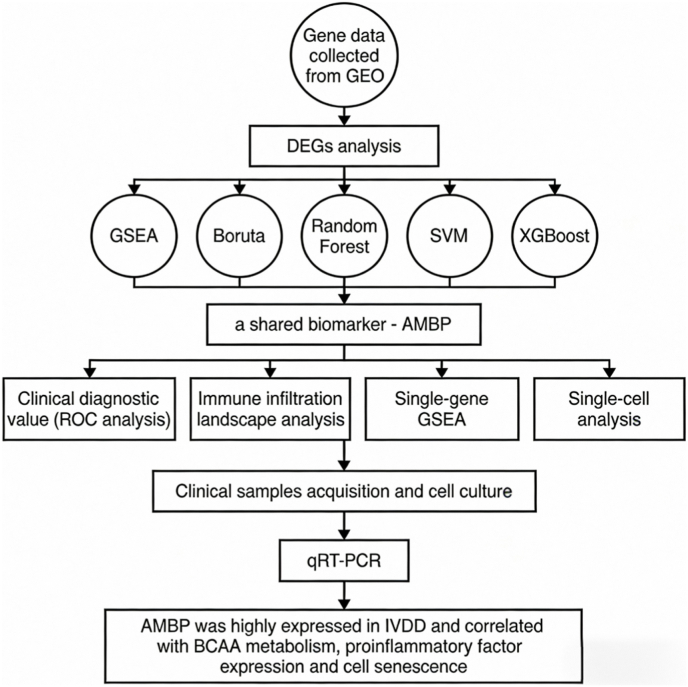
The entire flow chart of our research.

**Fig. 2:**
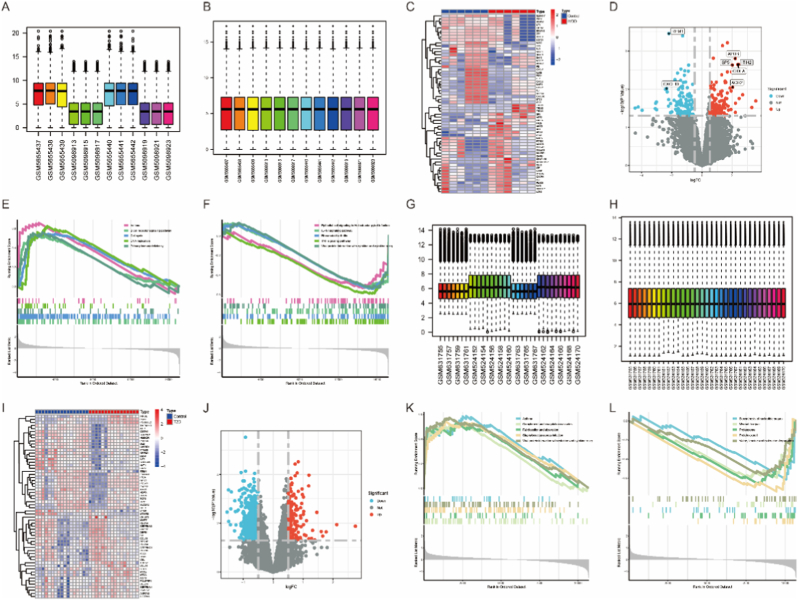
Processing and identification of differentially expressed genes in the IVDD and T2D datasets were conducted. (A, B) Box plots demonstrate the normalization process for the IVDD dataset. (C, D) Volcano plots and heatmaps depict the differential expression patterns of genes in the IVDD group. (E, F) GSEA analysis was performed to investigate enriched pathways associated with differentially expressed genes in the IVDD group. (G, H) Box plots display the normalization results for the T2D dataset. (I, J) Volcano plots and heatmaps illustrate the differential expression patterns of genes in the T2D group. (K, L) GSEA analysis was conducted to explore enriched pathways associated with differentially expressed genes in the T2D group.

**Fig. 3:**
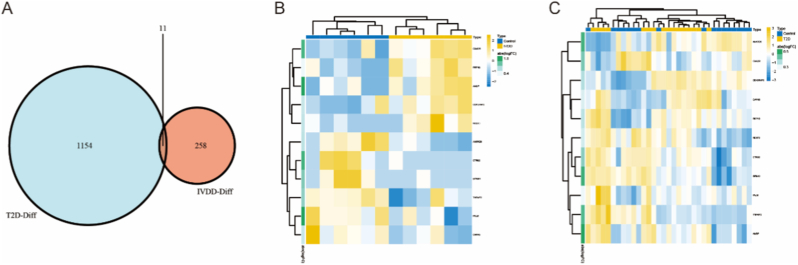
(A) A Venn diagram illustrates the 11 commonly differentially expressed genes between IVDD and T2D. (B) A heatmap displays the expression levels of these 11 genes in the IVDD group. (C) The expression levels of these 11 genes in the T2D group are visualized using a heatmap.

The process of the research is depicted in Fig. 1. We integrated and standardized the IVDD and T2D datasets, resulting in two merged datasets (Fig. 2A, B, G, H). The IVDD integrated dataset consisted of 6 control groups and 6 case groups, while the T2D integrated dataset included 17 control groups and 16 case groups. As depicted in Fig. 2A and B, standardization significantly reduced the discrepancies between datasets. Employing a cutoff of corrected p-value ≤0.05 and log2(fold change) ≥1 for differential expression analysis, we identified a total of 258 DEGs in the IVDD disease group and 1154 DEGs in the T2D disease group compared to controls. Volcano plots and heatmaps were utilized to visualize the expression patterns of DEGs in both merged datasets (Fig. 2C, D, I, J). Subsequently, GSEA was conducted on these sets of DEGs to identify enriched functional pathways (Fig. 2E, F, K, L). In IVDD, B-cell receptor signaling pathway, cell cycle, DNA replication, asthma and primary immunodeficiency are upregulated, but pathways like Epithelial cell signaling in *Helicobacter pylori* infection, IL-17 signaling pathway, Rheumatoid arthritis, TNF signaling pathway, and Viral protein interaction with cytokine and cytokine receptor are downregulated (Fig. 2E and F). Besides, pathways such as Asthma, Complement and coagulation cascades, Fat digestion and absorption, *Staphylococcus aureus* infection, and Viral protein interaction with cytokine and cytokine receptor are upregulated in T2D (Fig. 2K). Biosynthesis of nucleotide sugars, Mismatch repair, Proteasome, and Protein export pathways are downregulated in T2D (Fig. 2L). By merging these two sets of DEGs together, 11 genes were identified including CXADR, RRP1B, AMBP, CDK5RAP2, NDST3, ANKRD6, CTRB2, SPINK1, TNFAIP2, PALM, and CAPN5 (Fig. 3A). The heat maps were used to show the expression levels of 11 genes in the respective study cohorts (Fig. 3B and C).

### 3.2 Screening for potential biomarkers

**Fig. 4:**
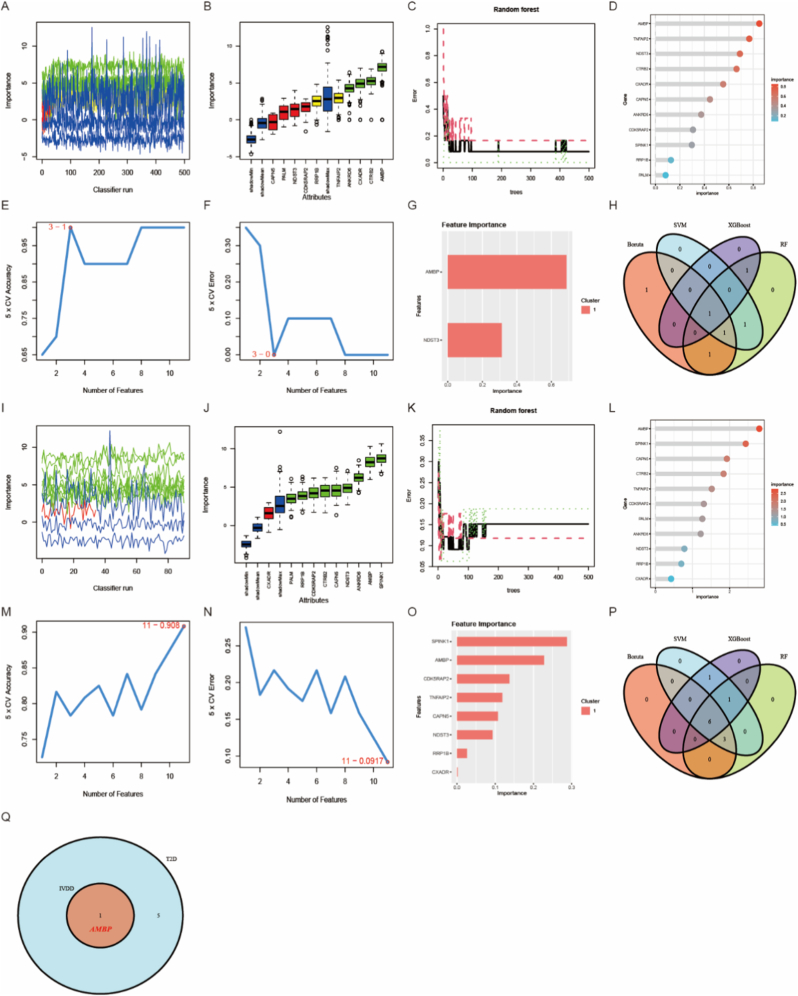
Screening of potential biomarkers for IVDD and T2D was conducted using four machine learning algorithms on 11 DEGs. (A, B) Boruta identified 4 feature genes in the IVVD group, with green indicating confirmed features, yellow indicating pending confirmation, red indicating rejected features, and blue representing shadow features. (C, D) RF selected 5 feature genes with importance greater than 0.5 in the IVDD group. (E, F) SVM identified 3 feature genes in the IVDD group. (G) XGBoost identified 2 feature genes for IVDD screening. (H) A Venn diagram illustrates the intersection of feature genes selected by these four algorithms in the IVDD group. (I, J) Using a similar color coding as mentioned earlier, the Boruta algorithm identified 9 feature genes in the T2D group. (K, L) RF selected 10 feature genes with importance greater than 0.5 in the T2D group. (M, N) SVM identified 11 feature genes for T2D screening. (O) XGBoost selected 8 feature genes for T2D screening. (P)Venn diagram shows the intersection of feature genes selected by these four algorithms in the T2D group. (Q)Venn diagram displays a potential biomarker-AMBP-for both IVDD and T2D.

In order to identify potential biomarkers for IVDD and T2D, we performed analyses using machine learning algorithms on the 11 differentially expressed genes. For IVDD, Boruta identified four feature genes including AMBP, CTRB2, CXADR, ANKRD6 (Fig. 4A and B). RF selected five feature genes (AMBP, TNFAIP2, NDST3, CTRB2, CXADR) with importance scores greater than 0.5 (Fig. 4C and D). SVM obtained three genes with the highest accuracy in a 3-point cross-validation and lowest error rate (Fig. 4E and F). XGBoost discovered two feature genes including AMBP and NDST3 (Fig. 4G). One intersection gene was obtained by combining the results of the four algorithms (Fig. 4H). In T2D, nine feature genes were identified by Boruta including RRP1B, AMBP, CDK5RAP2, NDST3, ANKRD6, CTRB2, SPINK1, PALM, and CAPN5 (Fig. 4I and J). Ten feature genes with importance scores greater than 0.5 were selected by RF including RRP1B, AMBP, CDK5RAP2, NDST3, ANKRD6, CTRB2, SPINK1, TNFAIP2, PALM, and CAPN5 (Fig. 4K and L). Eleven best-performing genes based on an-11 point cross-validation accuracy and lowest error rate were obtained through SVM (Fig. 4M and N). Eight feature genes were found by XGBoost algorithm including CXADR, RRP1B, AMBP, CDK5RAP2, NDST3, SPINK1, TNFAIP2, and CAPN5 (Fig. 4O). The intersection of all these algorithms yielded six common genes (Fig. 4P). By merging the intersecting gene sets from both diseases, we successfully identified AMBP as an ultimate potential biomarker for IVDD and T2D (Fig. 4Q). AMBP gene is located on chromosome 9 and encodes Alpha-1-Microglobulin (A1M) and Bikunin protein [31]. A1M is a member of the lipoprotein transport superfamily and plays an important role in the immune regulatory system. Bikunin, a serine protease inhibitor, is involved in the control of endothelial cell growth and extracellular matrix stability [31].

**Fig. 5:**
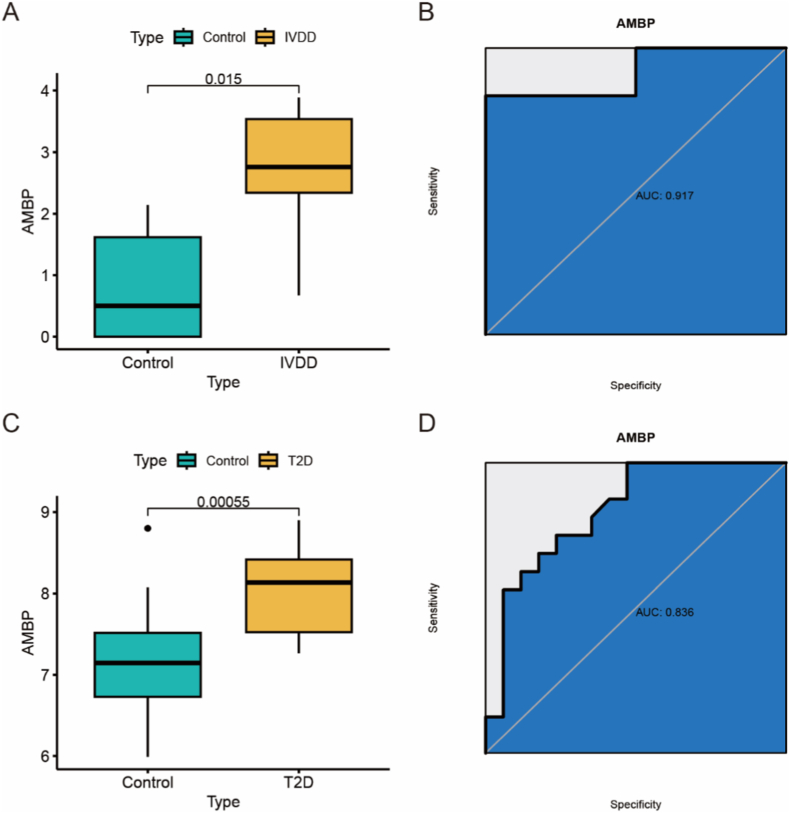
Expression levels and clinical diagnostic value analysis of AMBP in two diseases were investigated. (A) Box plots revealed significantly elevated expression of AMBP in IVDD. (B) The ROC curve for AMBP in the IVDD group demonstrated excellent clinical diagnostic value. (C) Box plots exhibited significantly higher expression of AMBP in T2D. (D) The ROC curve for AMBP in the T2D group indicated excellent clinical diagnostic value.

We conducted an analysis of AMBP expression patterns in IVDD and T2D to evaluate its diagnostic capabilities. The expression analysis revealed a significant upregulation of AMBP in both IVDD and T2D (Fig. 5A–C). Subsequently, we utilized ROC curves to assess the specificity and sensitivity of AMBP for diagnosing both conditions. The results from the ROC curves indicated that AMBP has excellent clinical diagnostic value for both diseases (Fig. 5B–D).

### 3.3 Immune infiltration analysis of biomarker

**Fig. 6:**
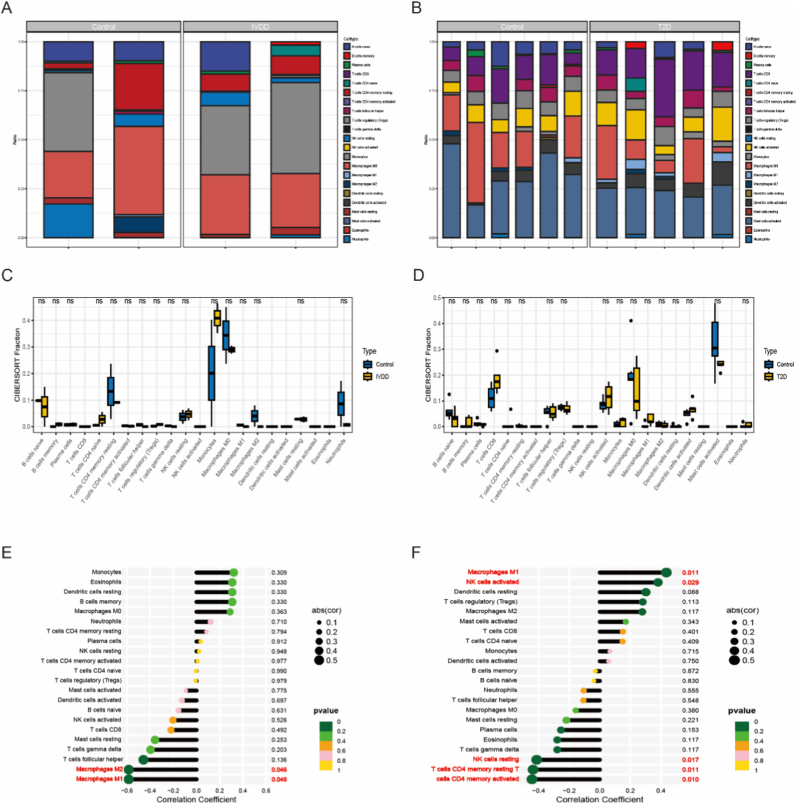
Analysis of immune cell infiltration in IVDD and T2D was conducted. (A) Stacked bar graphs were employed to depict the extent of immune cell infiltration in the IVDD group. (B) Stacked bar graphs were utilized to illustrate the degree of immune cell infiltration in the T2D group. (C) Violin plots were used to compare the expression levels of 22 types of immune cells between the IVDD and control groups. (D) Violin plots displayed differences in the expression levels of 22 types of immune cells between the T2D and control groups. (E) The relationship between AMBP expression and immune cells in the IVDD group was demonstrated. (F) Analysis of AMBP expression in immune cells from the T2D group was performed.

Immune cell infiltration profiles in IVDD and T2D samples versus controls were analyzed using the CIBERSORT algorithm. Fig. 6A and B illustrate the proportional distribution of 22 immune cell types in IVDD and T2D, respectively. Compared to controls, IVDD samples exhibited elevated naïve B and T cells, but reduced resting CD4^+^ memory T cells, M2 macrophages, and neutrophils (Fig. 6C). In T2D, memory B cells, activated NK cells, M0 and M1 macrophages increased, while activated mast cells decreased (Fig. 6D). Correlation analysis revealed a significant negative association between AMBP expression and M1/M2 macrophage ratios in IVDD (Fig. 6E). In T2D, AMBP positively correlated with M1 macrophages and activated NK cells, but negatively correlated with resting NK cells and memory T cell subsets (both resting and activated CD4^+^ T cells) (Fig. 6F). These results suggest that AMBP expression is associated with altered immune cell profiles in both IVDD and T2D, providing clues for its potential role in immunoregulation.

### 3.4 Single-gene GSEA

**Fig. 7:**
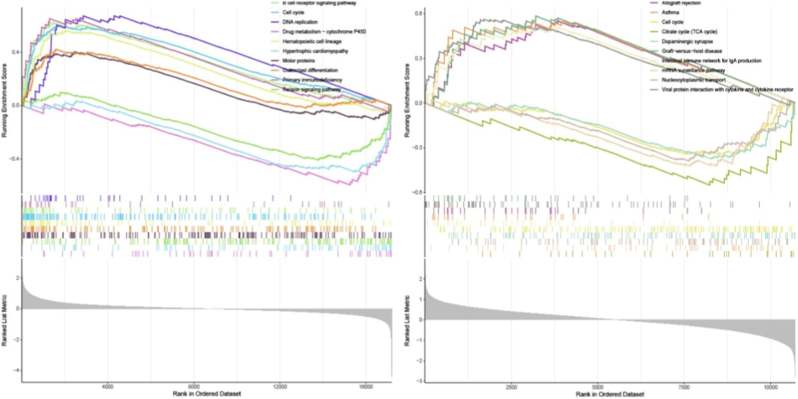
Single-gene GSEA analysis of AMBP. Left: IVDD, Right: T2D.

Subsequently, single-gene GSEA was conducted to investigate the biological functions and pathway enrichment of AMBP. Fig. 7 illustrates that AMBP plays a role in regulating the cell cycle and is enriched in various immune-related signaling pathways in both diseases. In IVDD, AMBP exhibits enrichment in immune-related pathways such as the B-cell receptor signaling pathway and primary immunodeficiency. In T2D, AMBP demonstrates enrichment in the Cell cycle, Asthma, and Dopaminergic synapse. Furthermore, upregulation of AMBP is observed in TCA cycle pathway in T2D, suggesting its involvement in cellular metabolism regulation.

### 3.5 Single-cell analysis

**Fig. 8:**
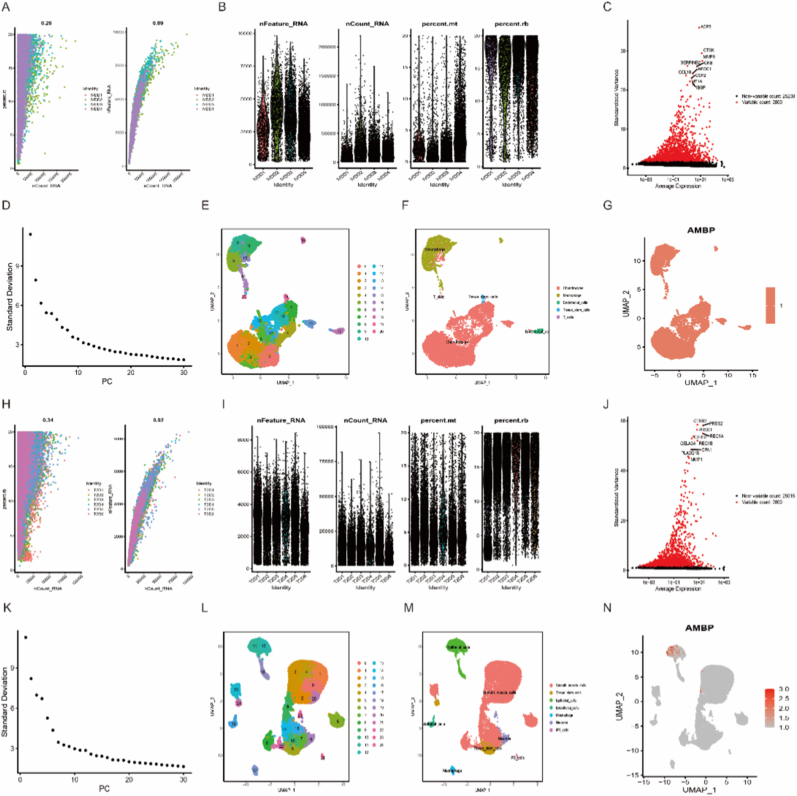
Single-cell sequencing analysis was conducted to investigate the role of AMBP in two diseases. In IVDD groups: (A) The correlation between gene count and feature genes was evaluated. (B) Mitochondrial and red blood cell genes were controlled to ensure sample quality. (C) 2000 highly variable genes were highlighted in red color with labels for the top ten most important genes. (D) The number of principal components was selected. (E) 21 subgroups of cells were identified, which were annotated as five immune cell clusters. (F) Significant expression of AMBP was marked in specific cell subgroups. (G) Significant expression of AMBP within specific cellular subgroups was marked. In T2D groups: (H) The correlation between gene count and feature genes was evaluated, while mitochondrial and red blood cell genes were controlled to ensure sample quality. (I) Mitochondrial and red blood cell genes were controlled to ensure sample quality. (J) 2000 highly variable genes using red color with labels for top ten most important genes were highlighted. (K) PC numbers were selected. (L) 21 cellular subgroups were identified, which are annotated as seven immune cell clusters. (M) Significant expression of AMBP was marked in specific cell subgroups. (N) Significant expression of AMBP within specific cellular subgroups.

The datasets of two different diseases were subjected to single-cell sequencing. Cells with mitochondrial gene expression >10% were excluded. The count relationship between samples and feature genes in IVDD and T2D was 0.89 and 0.92, respectively, indicating adequate sequencing depth (Fig. 8A, B, H, I). Subsequently, the top 2000 highly variable genes were selected for visualization in red color, and the top 10 important genes were identified (Fig. 8C–J). The number of PCs was determined based on Fig. 8D–K. Next, the UMAP algorithm was employed to classify individual samples. In IVDD, cells formed into 21 subclusters (Fig. 8E), with annotation revealing five immune cell subclusters (Fig. 8F). Similarly, in T2D, cells formed into 21 subclusters (Fig. 8L), annotated as seven immune cell subclusters (Fig. 8M). Finally, our analysis demonstrated significant expression of the biomarker AMBP across multiple cell subclusters in IVVD (Fig. 8G), while it predominantly expressed in epithelial cell subcluster in T2D (Fig. 8N). These findings from single-cell sequencing align with the results obtained from immune cell infiltration analysis mentioned earlier (Fig. 6), suggesting that AMBP may exert its influence on both diseases through immunoregulation.

### 3.6 Disorders of BCAA metabolism in patients with IVDD

**Fig. 9:**
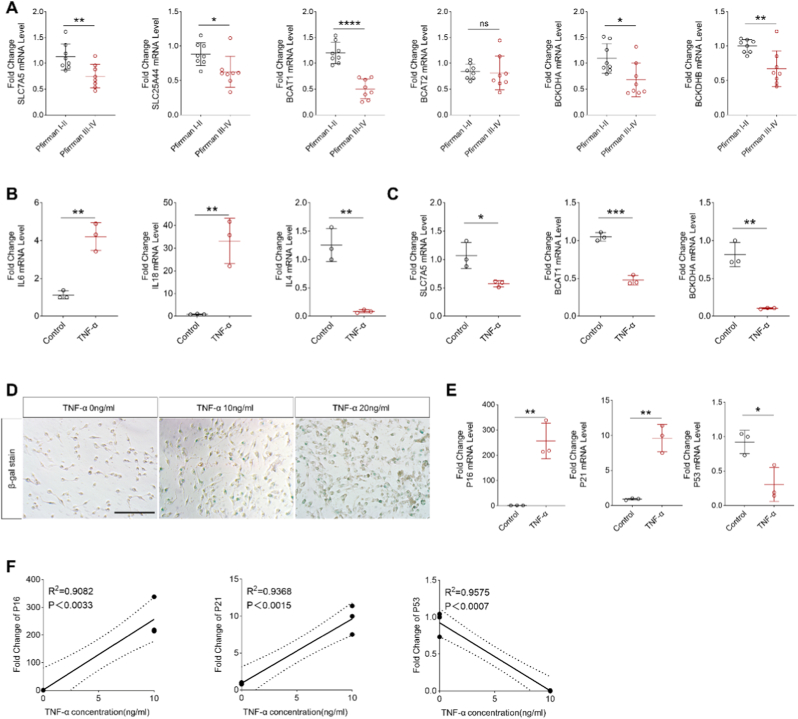
BCAA metabolism and immune changes in degenerated NP cells. (A) qRT-PCR was conducted to analysis the mRNA levels of BCAA metabolism-related enzymes and BCAA transporter in grade I/II and grade III/IV NP tissues; n = 20; P < 0.05. (B, C) Expression levels of mRNA for pro-inflammatory factors, BCAA metabolism-related enzymes, and BCAA transporters in control group and TNF-α-treated HNPC cells. (D) Expression of SA-β-gal in HNPC cells stimulated with different concentrations of TNF-α. (E, F) p16, p21, p53 mRNA level in TNF-α-stimulated HNPC cells,and correlation analysis with TNF-α concentration.

The metabolism of BCAAs is essential for normal physiological processes. To investigate the changes in BCAA metabolism in the nucleus pulposus (NP) cells of IVDD patients, we examined the mRNA of BCAA metabolism-related enzymes in the NP cells of IVDD patients. The results of PCR suggested that the transcription of BCAA metabolism-related enzymes decreased significantly as IVDD patients progressed (Fig. 9A). We found that the transcription of pro-inflammatory cytokines IL6 and IL19 was upregulated in myeloid cells after TNF-α stimulation. The transcription of the anti-inflammatory cytokine IL4 was downregulated (Fig. 9B). Transcription of BCAA metabolism-related enzymes was similarly downregulated (Fig. 9C). The results of SA-β-gal staining suggested that as the concentration of TNF-α in the culture medium rose, the expression of SA-β-gal in HNPC cells increased, the transcription of P16 and P21 increased, and the transcription of P53 decreased (Fig. 9D and E). The data from regression analysis also showed that the expression of P16 and P21 was positively correlated with the concentration of TNF-α while the expression of P53 was negatively correlated with the concentration of TNF-α (Fig. 9F).

**Fig. 10:**
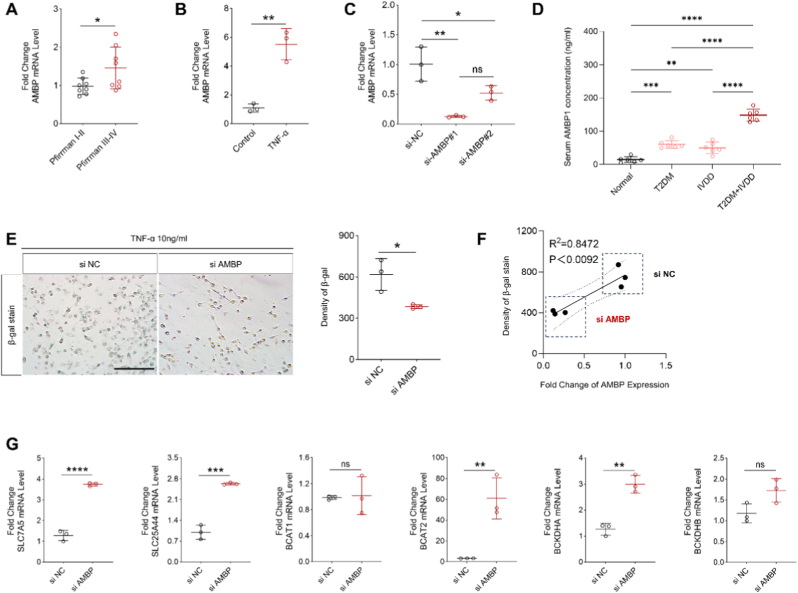
AMBP promotes imbalance of BCAA metabolism. (A) mRNA transcripts of AMBP in patients with different grades of IVDD and relative quantitative analysis. (B) mRNA transcripts of AMBP in TNF-α-stimulated HNPC cells and relative quantitative analysis. (C) Detection of inhibition efficiency of small interfering RNAs against different targets. (D) Serum concentrations of AMBP in normal, T2DM, IVDD, and T2DM combined with IVDD patients were examined using ELISA. (E) Representative β-gal staining images of HNPC cells before and after AMBP inhibition. (F) Correlation analysis between AMBP expression and β-gal expression. (G) mRNA transcripts of BCAA metabolism-related enzymes in TNF-α-stimulated HNPC cells before and after AMBP inhibition and relative quantitative analysis.

We next examined the expression of IVDD maker-AMBP predicted by bioinformatics analyses in IVDD patients as well as in HNPC after TNF-α stimulation. Consistent with the results predicted by the bioinformatics results, the expression of AMBP was progressively upregulated with the progression of IVDD patients, and the transcription of AMBP was also significantly higher in TNF-α-stimulated HNPC cells than in control HNPC cells (Fig. 10A and B). To clarify the link between AMBP and BCAA metabolism, we first tested the inhibitory efficiency of the two designed small interfering RNAs for AMBP in the HNPC cell line. We could identify that si-AMBP#1 had the best inhibition efficiency for AMBP in HNPC cells, for this reason we chose si-AMBP#1 for subsequent studies (Fig. 10C). Serum concentrations of AMBP in normal, T2DM, IVDD, and T2DM combined with IVDD patients were examined using ELISA, and the results suggested that AMBP was significantly elevated in patients with T2DM combined with IVDD (Fig. 10D). After we inhibited AMBP expression in HNPC cells using small interfering RNA, HNPC senescence induced by TNF-α was reversed, and the senescence level of HNPC was positively correlated with AMBP expression (Fig. 10E and F). Meanwhile, we discovered that TNF-α-induced impairment of BCAA metabolism in HNPC cells was also reversed (Fig. 10G).

**Fig. 11:**
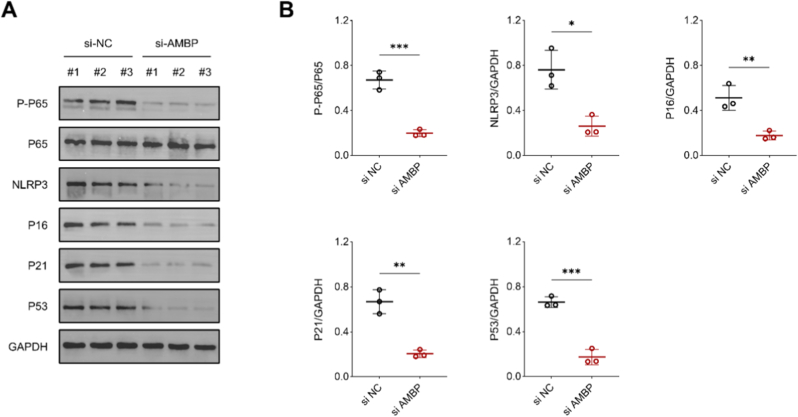
Knockdown of AMBP inhibits inflammation-induced aging. (A) Representative protein strips before and after AMBP inhibition. (B) The results of the semi-quantitative analysis of the protein strips and statistics.

After clarifying the function of AMBP, we examined the reasons why AMBP contributes to metabolic imbalance as well as aging. Upon knockdown of AMBP, the phosphorylation of P65 was decreased and the expression of NLRP3 was decreased, implying the suppression of intracellular inflammatory levels. Decreased levels of inflammation led to a decrease in aging MARKERS, as evidenced by decreased protein concentrations of P16, P21, and P53 (Fig. 11A and B).

## 4 Discussion

IVDD is a prevalent chronic musculoskeletal disorder worldwide and serves as the primary etiology of lower back pain, significantly impacting individuals' quality of life and overall societal well-being. T2D, being the most common metabolic disease, can contribute to the initiation and progression of IVDD through diverse mechanisms. The accumulation of advanced glycation end products within the nucleus pulposus during T2D and IVDD progression may impede Sirtuin3 (SIRT3) functionality and disrupt the mitochondrial antioxidant network, ultimately leading to nucleus pulposus cell apoptosis [32]. AGEs can also trigger inflammation in nucleus pulposus by activating NLPR3, thereby promoting the progression of intervertebral disc degeneration [19]. Hyperglycemia can induce apoptosis of cartilage endplate cells by up-regulating MALAT1 expression [33]. In rat NP cells, Glucose can also induce stress-induced senescence in a time- and dose-dependent way [34]. T2D can also induce microvascular lesions in cartilage endplate, resulting in narrowing and occlusion, which further impairs substance exchange between intervertebral disc cells and vertebral bodies. Consequently, this leads to inadequate nutrient supply for the nucleus pulposus and accumulation of toxic metabolites, ultimately causing increased cell death in NP. However, the underlying mechanisms and potential biomarkers associated with T2D-related IVDD remain incompletely understood. To our knowledge, our study is pioneering in exploring potential shared biomarkers between IVDD and T2D.

The present study demonstrates that AMBP, a potential biomarker for both IVDD and T2D, is significantly upregulated in both diseases and exhibits excellent clinical diagnostic value. By integrating the results of CYBERSORT and single-cell sequencing, it becomes evident that multiple immune cell infiltrations occur in IVDD and T2D. Also, the expression of AMBP exhibits a strong positive correlation with immune cells and is significantly upregulated in various subsets of immune cells. Single-gene GSEA analysis suggests the involvement of AMBP in regulating cell cycle progression as well as modulating multiple immune pathways in both diseases. In IVDD, AMBP enrichment is observed in immune-related pathways, notably B-cell receptor signaling pathway and primary immunodeficiency; whereas in T2D, AMBP enrichment occurs primarily within the chemokine signaling pathway, NF-kappa B signaling pathway, and autophagy vesicle formation process. Previous studies have demonstrated the pivotal role of immune infiltration in the pathogenesis of both IVDD and T2D. Three distinct macrophage markers were identified,and macrophage infiltration were observed within degenerated intervertebral disc cells [35,36]. Macrophage polarization exerts regulatory effects on cell proliferation, extracellular matrix metabolism, and inflammatory mediators, thereby influencing IVDD [37]. M1 macrophages play a pivotal role as the primary source of pro-inflammatory cytokines in the initiation and progression of pancreatic inflammation, while M2 macrophages are indispensable for pancreatic development and β-cell proliferation [38]. The production of inflammatory mediators by adipocytes and macrophages in the adipose tissue of individuals with diabetes mellitus can induce chronic inflammation in T2D, leading to β-cell damage and inadequate insulin secretion, ultimately resulting in hyperglycemia [39]. Furthermore, inflammation may contribute to the development of T2D through insulin resistance [40]. In conclusion, our research provides preliminary evidence suggesting a potential link between AMBP and immune microenvironment changes in IVDD and T2D, which warrants further investigation.

BCAA has emerged as a prominent focus in T2D research in recent years and is regarded as one of the most robust biomarkers for obesity, insulin resistance, T2D, and cardiovascular diseases [22,41,42]. BCAA and BCKA have the ability to activate mammalian mTORC1, leading to the inhibition of insulin signaling, while inhibitors of BCKDH kinase demonstrate significant potential in alleviating high-fat diet-induced insulin resistance in mice [43]. However, to the best of our knowledge, there is currently a dearth of research investigating the role of BCAA metabolism in IVDD. Therefore, we conducted *in vitro* experiments and found that the expression levels of BCAA metabolism-related enzymes decreased as IVDD progressed, while knocking down AMBP reversed this outcome. This suggests that AMBP may be associated with the downregulation of BCAA metabolism-related enzymes observed in IVDD.

The single-gene GSEA results also revealed that AMBP exhibits enrichment in the TCA cycle. The TCA cycle, as the center of cellular metabolism, plays an important role in governing DNA methylation, chromatin modification, and post-translational modifications of proteins. Moreover, it exerts critical regulatory control over tumor development, stem cell functionality, immune responses, and various other cellular signaling pathways [44]. Metabolites of BCAA such as acetyl coenzyme A and succinyl coenzyme A can serve as substrates for both the TCA cycle,and release adenosine triphosphate to provide energy for cells [24]. Previous studies have demonstrated impaired flux of the TCA cycle in individuals with T2D, which serves as both a determinant and an indicator of the T2D phenotype [45]. Impaired flux of the TCA cycle hampers mitochondrial oxidative phosphorylation, resulting in diminished ATP production and heightened ROS generation. Consequently, this exacerbates mitochondrial dysfunction and accelerates cellular senescence [46]. In addition, our experimental results confirm that the knockout of AMBP reduces the aging phenotype in degenerated HNPC cells (Fig. 9D/10E). Thereby, we hypothesize that AMBP might be linked to impaired tricarboxylic acid cycle flux potentially through its association with BCAA metabolism, which could contribute to NP cell senescence.

However, our study has several limitations that should be acknowledged: (1) The sample size of the GEO datasets, particularly for IVDD (6 controls vs. 6 cases), is relatively small, which may affect the statistical power and generalizability of our bioinformatics findings. Our study should therefore be considered exploratory. (2) The current evidence primarily establishes correlations. While the *in vitro* knockdown experiment suggests a functional role, the precise molecular mechanism by which the secreted protein AMBP influences intracellular BCAA metabolic gene transcription remains unclear. Future studies are needed to determine whether this occurs through receptor-mediated signaling or other pathways. (3) Our *in vitro* model used TNF-α to simulate a chronic inflammatory microenvironment common to both diseases. To more specifically model the hyperglycemic condition of T2D, future studies should incorporate high glucose treatment. (4) Assessment of BCAA metabolism was limited to mRNA levels of related enzymes; direct measurement of metabolic flux or BCAA metabolite concentrations would provide stronger mechanistic insight. Our research identifies AMBP as a shared biomarker with diagnostic potential for IVDD and T2D, reveals its correlation with immune landscape alterations and BCAA metabolism, and suggests it as a candidate regulatory factor worthy of further investigation.

## List of abbreviations

IVDD: Intervertebral disc degeneration; T2D: Type 2 diabetes; GEO: Gene Expression Omnibus; DEGs: differentially expressed genes; RF: Random Forest; SVM: Support Vector Machine; XGBoost: Extreme Gradient Boosting; NP: nucleus pulposus; AF: annulus fibrosus; CEP: cartilaginous end plate; AGEs: Advanced glycation end products; BCAA: Branched-chain amino acid; DEGs: differentially expressed genes; GSEA: Gene set enrichment analysis; qRT-PCR: Quantitative Real-Time Polymerase Chain Reaction; ELISA: Enzyme-linked immunosorbent assay; SA-β-gal: Senescence-Associated β-Galactosidase; A1M: Alpha-1-Microglobulin; NP: nucleus pulposus.

## Human ethics and consent to participate declarations

The studies involving human participants were reviewed and approved by the Ethics Committee of Second Affiliated Hospital of Naval Medical University. The patients provided their written informed consent to participate in this study. The studies followed the guidelines outlined in the Declaration of Helsinki.

## Animal ethical statement

All animal experiments were conducted in compliance with the ARRIVE guidelines, and were carried out in accordance with the U.K. Animals (Scientific Procedures) Act, 1986, accordance with the National Institutes of Health Guide for the Care and Use of Laboratory Animals (NIH Publications No. 8023, revised 1978). All animal experiments conformed to the internationally accepted principles for the care and use of laboratory animals.

## Consent for publication

Written informed consent for publication of anonymized data was obtained from all participants.

## Funding

This research was supported by the 10.13039/501100012166National Key R&D Program of China (Key Special Project for Marine Environmental Security and Sustainable Development of Coral Reefs 2022-3.5) and 10.13039/501100001809National Natural Science Foundation of China (No.82102605).

## CRediT authorship contribution statement

**Ba Qiu:** Conceptualization, Data curation, Methodology, Visualization, Writing – original draft, Writing – review & editing. **Tao Chen:** Formal analysis, Methodology, Visualization, Writing – original draft, Writing – review & editing. **Qi Hong:** Conceptualization, Data curation, Formal analysis, Validation, Visualization, Writing – review & editing. **Xiaoxing Xie:** Conceptualization, Data curation, Formal analysis, Investigation, Methodology, Software, Validation. **Mintao Xue:** Conceptualization, Data curation, Formal analysis, Investigation, Methodology, Validation, Visualization. **Ning Xu:** Conceptualization, Formal analysis, Methodology, Software, Validation, Visualization. **Changgui Shi:** Investigation, Methodology, Validation, Visualization, Writing – original draft, Writing – review & editing. **Guohua Xu:** Conceptualization, Data curation, Formal analysis, Investigation, Methodology, Software. **Weiheng Wang:** Conceptualization, Data curation, Formal analysis, Investigation, Software, Visualization. **Muhammad Sulaiman:** Conceptualization, Data curation, Formal analysis, Software, Validation. **Yanhai Xi:** Funding acquisition, Investigation, Methodology, Project administration, Resources, Software, Supervision, Validation.

## Declaration of competing interest

The authors declare that they have no competing interests.

## Data Availability

Data will be made available on request.
